# Monitoring Junction Temperature of RF MOSFET under Its Working Condition Using Fiber Bragg Grating

**DOI:** 10.3390/mi13030463

**Published:** 2022-03-18

**Authors:** Zhenmin Liu, Na Chen, Yong Liu, Zhenyi Chen, Fufei Pang, Tingyun Wang

**Affiliations:** The Key Laboratory of Specialty Fiber Optics and Optical Access Networks, Joint International Research Laboratory of Specialty Fiber Optics and Advanced Communication, Shanghai University, Shanghai 200444, China; liuzhenmin@shu.edu.cn (Z.L.); liu_yong@shu.edu.cn (Y.L.); zychen@shu.edu.cn (Z.C.); ffpang@shu.edu.cn (F.P.); tywang@shu.edu.cn (T.W.)

**Keywords:** fiber gratings, junction temperature, optical fiber sensors, radio frequency, MOSFETs

## Abstract

When a high-power radio frequency (RF) metal oxide semiconductor field effect transistor (MOSFET) works in low-efficiency situations, considerable power is dissipated into heat, resulting in an excessive junction temperature and a likely failure. In this study, an optical fiber Bragg grating (FBG) sensor is installed on the die of a high-power RF MOSFET. The temperature change of RF MOSFET with the change of input signal is obtained by using the temperature frequency shift characteristic of the FBG reflected signal. Furthermore, the fast and repetitive capture of junction temperature by FBG reveals details of the temperature variation within each RF pulse, which is correctly correlated with input signals. The results show that besides monitoring the temperature accumulation of the chip for a long time, the FBG can also capture junction temperature details of the chip within each pulse period. Finally, a Cauer-type thermal model of the RF MOSFET was constructed based on the temperature information captured by the FBG.

## 1. Introduction

A radio frequency (RF) metal oxide semiconductor field effect transistor (MOSFET) is widely used to amplify pulsed-RF signals in applications such as aerospace, radar, magnetic resonance imaging (MRI), etc. [1,2,3]. For most application scenarios, the reliability and stability of the MOSFETs are important bases for evaluating the performance of the device. However, because the operating efficiency of the high-power RF MOSFETs is much less than one, high-power RF MOSFETs inevitably generate excess heat during the working process, resulting in an excessive junction temperature, which may trigger failure in thermal management [4]. In other words, the junction temperature has become one of the barriers for the continued development of this technology. Therefore, an accurate, rapid, and real-time method for monitoring the thermal operating status of the junction temperature of MOSFETs will be a powerful tool to facilitate the thermal management of such devices.

The rapid change in junction temperature and the strong electromagnetic interference of high-power RF MOSFETs during operation form a harsh temperature measurement environment [5]. It is impractical to use conventional metallic thermocouples for junction temperature measurements because the high level of electromagnetic interference can interfere with the measurement results and the metallic thermocouple can affect the circuit operating conditions. To avoid interference with the circuit, the junction temperature can be inferred by measuring thermally sensitive parameters such as the voltage drop, short-circuit current, and drive voltage of the device under test (DUT) [6,7,8,9,10]. However, this method is not capable of measurement while the DUT is in operation. Infrared thermometry can obtain the temperature distribution on the surface of the DUT without contact [11,12]. However, its continuous measurement is constricted by the system’s image sampling rate and image processing means [13]. Junction temperature estimation methods based on equivalent thermal network models are often used in real applications [14,15,16,17,18,19]. This method ensures that the thermal models derived from these samples can be applied to other devices of the same type by making accurate measurements of one or more semiconductor devices. However, the accuracy of the model is based on the modeling parameters measured in the experiment. For such a demanding measurement environment, the use of fiber optic sensors with good electromagnetic compatibility (EMC) and high acquisition rates will be a suitable solution [20]. Among them, fiber Bragg grating (FBG) has good stability, a large temperature measurement range, and excellent EMC [21,22,23,24]. It can be installed very close to the die when the chip is under its operation conditions. This has been proved in the scenario of IGBT junction temperature monitoring during its operation [25,26,27]. Therefore, for the RF MOSFET that works at a higher frequency and has more stringent requirements for electromagnetically compatible environments, the FBG’s effectiveness for junction temperature monitoring is worth evaluating.

This study designed MOSFET application scenarios and experiments to verify the FBG’s ability to rapidly capture thermal pulses under RF operating conditions, and to build thermal models based on the measurements.

## 2. Experimental Setup

**Figure 1:**
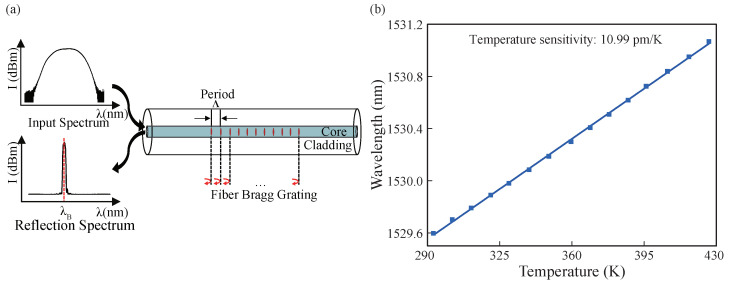
(**a**) Light propagation of FBG. (**b**) Relationship between $\lambda_{B}$ and temperature.

The whole experimental system includes FBG signal detection and RF circuit. The FBG signal detection system mainly consists of a FBG sensor, an optical interrogator (Micron Optics-sm130, which has a demodulation accuracy of 5 pm in the wavelength range of 1510∼1590 nm), and a personal computer (PC). The sensor is made by commercial FBG after temperature calibration in our lab. The principle of FBG and its temperature character is shown in Figure 1. It involves a periodic refractive index modulation along the core of an optical fiber, and it operates as an in-fiber band reject filter that reflects a narrowband optical signal at a particular wavelength called the Bragg wavelength $\lambda_{B}$. The light propagation along the FBG is illustrated in Figure 1a. The Bragg wavelength, $\lambda_{B}=2\cdot n_{eff}\cdot \Lambda$, is related to the grating pitch Λ and the effective refractive index $n_{eff}$ of the guided mode in the single mode fiber [28,29]. Due to the thermo-optical effect and the thermal expansion characteristics, both $n_{eff}$ and Λ change with the ambient temperature changes which lead to the shifts of the reflection spectrum of FBG that can be detected by the interrogator. Figure 1b shows the relationship between the $\lambda_{B}$ sampled by the interrogator and the corresponding temperature to FBG. In the calibration experiments, the slope of the linear fit is 10.99 ± 0.073 pm/K and the coefficient of determination is 0.99938. Taking into account the error factor brought by the demodulator and FBG sensor, the error of the whole measurement system is within ±0.5 K.

**Figure 2:**
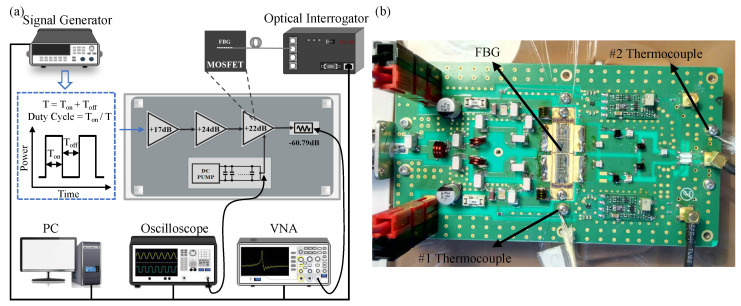
(**a**) Experimental system diagram. (**b**) Circuit module.

To demonstrate its ability for RF pulse detection, a three-stage RF amplifier was built with a high-power RF MOSFET module MRFE6VP61K25H, manufactured by NXP, as the DUT. The experimental set up is illustrated in Figure 2a. DUT plays a role as the final stage in a three-stage RF amplifier. Other parts of the RF circuit are composed of vector network analyzer (VNA), signal generator, direct current (DC) power supplier, capacitor bank, microwave attenuator, and the FBG sensor interrogating subsystem. Here, the three-stage RF amplifier works in a pulsed mode, and the signal generator is used to adjust the input signal duty cycle and pulse width to control the operating state of RF MOSFET. In addition, a trigger signal is used to establish the DC bias in the amplifier at all stages, and another synchronized trigger signal is used to activate the internal receiver of the VNA. The total gain of the three-stage amplifier is 63 dB, i.e., a 2 kW pulse power output is expected if 1 mW (0 dBm) is fed to the first stage. The high-power RF pulse is attenuated by 60.79 dB, including cable loss, before it is measured by the VNA. During the pulse-on period, the DC power supplier and a capacitor bank are used to provide enough energy and to maintain the DC voltage. The DC voltage and DC current are measured using an oscilloscope with voltage and current probes, respectively.

In order to directly detect the die temperature of the DUT, its ceramic cap was removed beforehand, and the FBG sensor was directly attached to the die using an optical UV adhesive with low shrinkage. As the temperature changes, the optical interrogator exhibits a change in wavelength once every 1 ms, i.e., the frequency of the thermal sample is 1 kHz. The electrical performance of the DUT was measured and recorded before the installation of FBGs, and it was compared with test results under the same conditions after installation. Owing to the excellent EMC capability of the FBG sensor, this comparison did not show any change in performance, thus verifying the advantage of using FBG. In addition, for comparison, the two thermocouples (Thermocouple 1 and Thermocouple 2) were respectively fixed near the chip flange, as shown in Figure 2b. These thermocouples were connected to a multi-channel thermocouple reader that updates and stores temperature data every few seconds. All instruments were programmed with appropriate configurations using a GPIB or LAN connection to PC. The time synchronization of all instruments and multi-measurement execution were driven by codes on the PC.

## 3. Results and Discussion

The experimental results show that the final amplifier has an efficiency of about 60%. Besides the output RF power, the rest of the input DC energy is dissipated by the chip into heat. Consequently, during the chip operation in pulsed-mode, “thermal pulses" will be generated over time, which trigger a rapid rise in DUT junction temperature. It should be noted that the first- and second-stage amplifiers also experience heat dissipation, which can be reasonably ignored compared to the final stage. The following analysis of experiment results together with dedicated conditions of input pulse will demonstrate the FBG thermal sensing technique for junction temperature.

### 3.1. The $\Delta T_{j}$ within One Pulse Period

**Figure 3:**
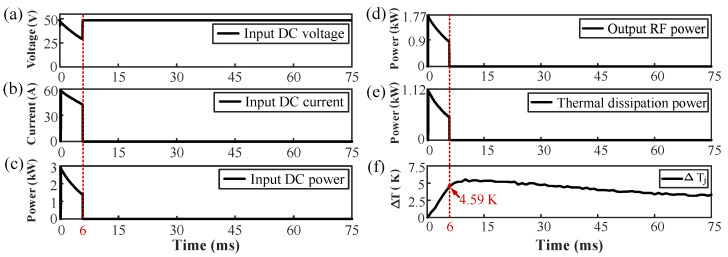
(**a**) Waveform of input DC voltage. (**b**) Waveform of input DC current. (**c**) Waveform of input DC power. (**d**) Waveform of output RF power. (**e**) Waveform of thermal dissipation power. (**f**) Waveform of junction temperature variation within 75 ms.

In these experiments, a pulse period is 75 ms and the duty cycle is set to 8%. During a pulse period, the input DC voltage, input DC current, output RF power, and DUT junction temperature of the amplifier were measured by oscilloscope with voltage and current probes, VNA, and interrogator, respectively. The difference between the input DC power and the output RF power is the thermal dissipated power generated by DUT. In addition, all experimental data are collected and processed by the PC, and the final result is shown in Figure 3. The experimental data show that the maximum input DC voltage, maximum input DC current, maximum input DC power, maximum output RF power, and maximum thermal dissipation power of the amplifier are 48.64 V, 59.47 A, 2.89 kW, 1.77 kW, and 1.12 kW, respectively.

As can be observed in Figure 3a, the input DC voltage is not ideally “flat-topped”, but has a linear downtrend at the top of the pulse, as with the input DC current in Figure 3b. The voltage drop during the pulse-on period is mainly caused by the capacitor bank, and the capacitor discharge voltage follows an exponential attenuation curve. Once the capacitor bank voltage is lower than 50 V, the DC power supplier starts to charge the capacitor bank. Since the charging current of the capacitor bank is much smaller than its discharging current, a large pulse current output can be obtained by combining a DC power supply with a low current output and the capacitor bank. Here, there are two reasons for the decrease in the output RF power: first, the output voltage of the capacitor bank is decreased; and secondly, the decrease in the amplification factor is caused by the rise in the junction temperature of the MOSFET. As shown in Figure 3f, when the electrical pulse of 6 ms ended, the junction temperature variation of the DUT increased to 4.59 K. Within 6 ms to the end of the pulse, there was a slight temperature rise before the temperature began to drop gradually. This occurred because of the distance between the temperature-sensitive part of the sensor and the die (about 60 μm, i.e., the thickness of the fiber cladding), and also because the sampling frequency of the fiber interrogator was limited.

**Figure 4:**
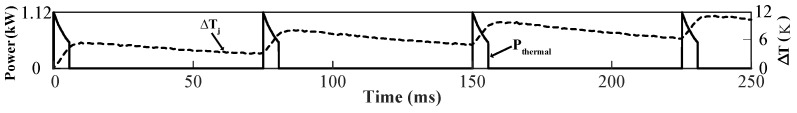
Junction temperature variation and thermal dissipation power of the amplifier within 250 ms.

Furthermore, the $\Delta T_{j}$ within 250 ms of the continued pulse is shown in Figure 4, and it can be observed that the FBG sensor can monitor both the junction temperature within each input signal pulse and the cumulative temperature during the working process. This result shows that the FBG sensor accurately captured and presented “temperature pulses” corresponding to an input electrical pulse.

### 3.2. Condition of Same Pulse Width and Different Duty Cycle

**Figure 5:**
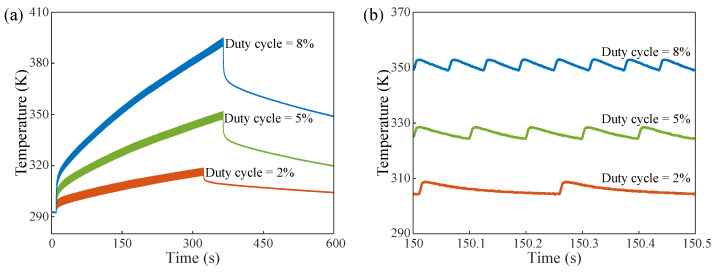
(**a**) The temperature variation measured by FBG with a pulse width of 5 ms and a duty cycle of 2%, 5%, and 8%, respectively. (**b**) The temperature variations measured by the FBG over 500 ms for a pulse width of 5 ms and a duty cycle of 2%, 5%, and 8%, respectively.

The total duration of the input RF pulse burst was about 300 s. After the signal source was shut down, the optical interrogator remained in the state of data collection for 600 s. As shown in Figure 5a, the temperature varied at a duty cycle of 2%, 5%, and 8% with a pulse width of 5 ms for the input signal. As can be observed from the overall trend of the change, when the circuit system enters the working state, the junction temperature rises suddenly from ambient temperature and continues to increase. When the circuit stops working, the junction temperature drops abruptly and slowly returns to ambient temperature. Moreover, under the condition of the same pulse width, the duty cycle increases, the junction temperature rises faster, and finally the accumulated temperature increases. This occurs because the more effective the pulses that are introduced to the system, the higher the average power of the chip, and the greater total energy converted to thermal energy within the same observation period. Similarly, the temperature curve within 500 ms after 150 s is created via local analysis, as shown in Figure 5b. Taking the signal of 150 s as an example, it can be clearly seen that the junction temperature rises with the increase in the duty cycle. Furthermore, the periods of the thermal pulses in Figure 5b coincide with the periods of input RF pulses with the corresponding duty cycle. This validates that the FBG sensor captures the junction temperature of the MOSFET.

### 3.3. Condition of Same Duty Cycle and Different Pulse Width

**Figure 6:**
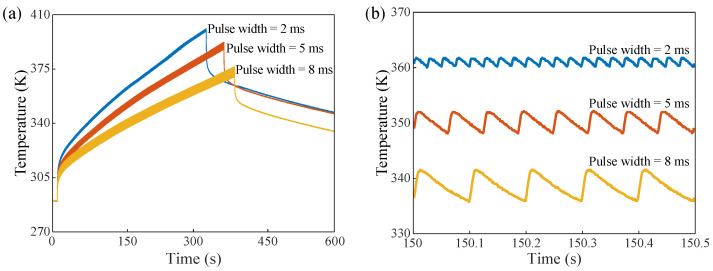
(**a**) The temperature variation measured by FBG with a duty cycle of 8% and pulse widths of 2 ms, 5 ms, and 8 ms, respectively. (**b**) The temperature variations measured by the FBG for 500 ms with a duty cycle of 8% and pulse widths of 2 ms, 5 ms, and 8 ms, respectively.

To ensure that the total energy input is constant, the duty cycle of the input signal was fixed to 8%, and the pulse width of the signal was changed to 2 ms, 5 ms, and 8 ms, respectively. The temperature measured by the FBG sensor is shown in Figure 6a. Similarly, it can be observed from the overall analysis that an increase in the pulse width of the input signal reduces the cumulative temperature. Theoretically, since the total energy of the input signal remains the same, the DUT should raise the same temperature in different pulse periods. However, the input pulse is not the ideal “flat top" pulse (refer to Figure 4). For example, if the pulse width is 2 ms and 5 ms, respectively, for non-standard rectangular pulses, the wider the pulse width, the greater the top drop of the pulse. Considering the chip, in the pulse-on state, the average power of the 2 ms pulse width is higher than that of the 5 ms pulse width, and the final curve shows that the cumulative temperature of the 2 ms is higher than that of the 5 ms within the same time. Moreover, when the duty cycle is fixed, the pulse width becomes wider, the pulse period is longer, and the temperature rise of the chip is higher in one period. This can be observed in Figure 6a: the wider the pulse width, the wider the curve. The time within 500 ms after 150 s is taken as an example for local analysis, as shown in Figure 6b.

### 3.4. Comparison between the FBG Sensor and Thermocouple and the Deduced Thermal Model

**Figure 7:**
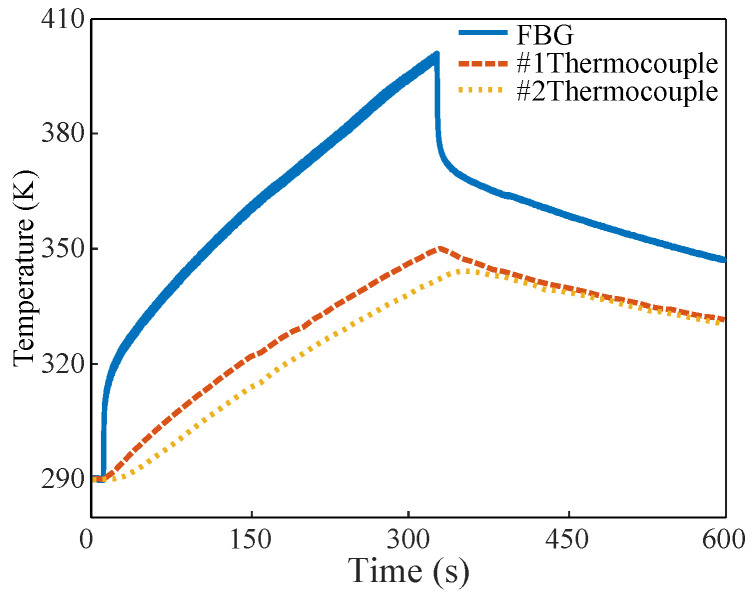
Comparison between the temperature measured by FBG and the temperature measured by the two thermocouples.

For the comparison, we took an input pulse width and duty cycle of 2 ms and 8%, respectively, as an example. The results of the temperature measured by the FBG sensor and that measured by two thermocouples are shown in Figure 7. Since the RF MOSFET has a high operating frequency and is sensitive to distributed capacitor and inductance, metal thermocouples should not be placed too close to the die, for the sake of security and circuit performance. The data of two thermocouples were collected by the Stanford Research System’s 16-channel thermocouple recorder, and the recorder collected data every few seconds, which is far below the Nyquist sampling frequency for RF signal of 128 MHz. According to the monitoring results, the overall trend in the change in the monitoring data of the two thermocouples is consistent with that of the FBG sensor. However, the rising trends of the temperature measured by the two thermocouples are much lower than that of the sensor, and the highest measured temperature of the two thermocouples is also much lower than that of the sensor. Additionally, the FBG sensor responds sensitively to temperature drops when the circuit stops working, whereas the response of the thermocouple is much slower. Therefore, it cannot reflect the actual junction temperature of the chip by measuring the temperature near the chip or outside the chip package.

**Figure 8:**
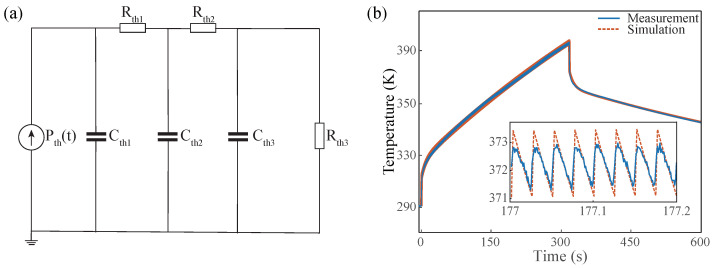
(**a**) The Cauer network thermal model of RF MOSFET circuit. (**b**) Comparison of thermal model output and FBG-measured temperature results.

As mentioned earlier in the paper, the thermal model of the RF MOSFET circuit can be obtained by fitting the temperature profile obtained from the FBG using the thermal model equivalent circuit method, where the thermal model is described using the thermal resistance $R_{th}$ in K/W and the thermal capacity $C_{th}$ in J/K. As shown in Figure 8a, the heat flow transfer model of the RF MOSFET circuit is built with a third-order Cauer network RC thermal model in this study, where the $P_{th}\left(t\right)$ denotes thermal pulse source with power [30].

Furthermore, the RF MOSFET circuit thermal model was obtained by fitting the third-order model to the data from FBG, and the parameters of the Cauer network thermal model are shown in Table 1. The MOSFET heat generation time coincides with the time when it is in operation; the output pulse of $P_{th}\left(t\right)$ can thus be set to a period of 25 ms, with a duty cycle of 8% and a peak of 1.17 kW. The Cauer network thermal model was simulated using the commercial software LTspice in combination with the above parameters. The simulation results are compared with the experimental results as shown in Figure 8b. The experimental data obtained by FBG as shown in the figure fit well with the simulation results. Therefore, the thermal model based on FBG measurement data is well able to simulate the temperature change of RF MOSFET circuit during operation.

## 4. Conclusions

In this study, the change in the junction temperature of a high-power RF MOSFET was monitored by bringing the FBG sensor in direct contact with the die. A RF MOSFET has a complex strong local electromagnetic environment, and it is very sensitive to distributed capacitance and inductance for the right impedance matching to maintain proper operating conditions. Although this method requires unpacking the IC packaging in the proof of concept, it provides very high speed and accurate measurements, compared to other technologies. The FBG sensors have good EMC characteristics. When the chip is working, direct contact with the die will not change the electrical characteristics of the chip. The operating efficiency of the amplifier is significantly less than one. When the amplifier is in operation, the electrical power dissipated is converted to thermal energy, resulting in an abrupt rise in junction temperature. For the chip in the pulse mode, FBG can accurately capture the “thermal pulses” generated by the electrical pulses introduced to the system, and the period of the thermal pulses sampled by the sensor corresponds to the period of the input RF pulses, such that FBG can precisely and sensitively measure the junction temperature of the chip. The temperature acquisition frequency in this study is 1 kHz, which depends on the sampling frequency of the optical interrogator. Furthermore, the experimental results show that the temperature near the chip does not reflect the real junction temperature of the chip, and it is not recommended to directly place a thermocouple on the die. Compared with the indirect measurement or estimation method, an FBG-sensor-based technology for direct measurement can swiftly, directly, and truly reflect the junction temperature of power devices, and also has potential advantages for monitoring the junction temperature of IGBT chips in real time and under high-speed switching conditions. Furthermore, this method can precisely identify the thermal characteristics of RF MOSFET and other semiconductor devices, which is constructive for the establishment of accurate models to simulate the heat generated during chip operation.

## Figures and Tables

**Table 1:** The parameters of the Cauer network thermal model.

$R_{th1}$	$R_{th2}$	$R_{th3}$	$C_{th1}$	$C_{th2}$	$C_{th3}$
0.259 K/W	0.232 K/W	3.213 K/W	0.725 J/K	50.312 J/K	205.125 J/K
